# Diode laser surgery in the treatment of oral proliferative verrucous leukoplakia associated with HPV-16 infection

**DOI:** 10.1186/s40902-018-0156-2

**Published:** 2018-07-30

**Authors:** Gian Paolo Bombeccari, Umberto Garagiola, Valentina Candotto, Francesco Pallotti, Francesco Carinci, Aldo Bruno Giannì, Francesco Spadari

**Affiliations:** 1 Maxillo-Facial and Dental Unit, Fondazione IRCCS Ca’ Granda Ospedale Maggiore Policlinico Milan, Department of Biomedical, Surgical, and Dental Sciences, University of Milan, Via Della Commenda 10, 20122 Milan, Italy; 2 Unit of Anatomical Pathology, Fondazione Ca’ Granda IRCCS Ospedale Maggiore Policlinico, University of Milan, Milan, Italy; 3 Department of Morphology, Surgery and Experimental Medicine, University of Ferrara, Ferrara, Italy

**Keywords:** HPV infection, Proliferative verrucous leukoplakia, Oral cancer, Diode laser

## Abstract

**Background:**

Proliferative verrucous leukoplakia (PVL) is an oral potentially malignant disorder, characterized by multifocal expression, progressive clinical evolution, and a high rate of malignant transformation. Evidence-based information regarding optimal PVL management is lacking, due to the paucity of data. The present report describes a case of PVL associated with HPV-16 infection and epithelial dysplasia treated by diode laser surgery, and the outcome of disease clinical remission over a 2-year follow-up period.

**Case report:**

A 61-year-old Caucasian male with oral verrucous hyperkeratosis presented for diagnosis. The lesions were localized on the maxillary gingiva and palatal alveolar ridge. Multiple biopsy specimens have been taken by mapping the keratotic lesion area. Microscopic examination was compatible with a diagnosis of PVL with focal mild dysplasia, localized in the right maxillary gingiva. Polymerase chain reaction (PCR) was done for human papillomavirus (HPV) detection which revealed presence of HPV DNA, and the genotype revealed HPV 16 in the sample. The PVL in the right gingival area was treated on an outpatient basis by excision with a diode laser. This approach resulted in good clinical response and decreased morbidity over a 2-year follow-up period.

**Conclusions:**

This case illustrates the benefit of a conservative approach by diode laser treatment than wide surgical excision for management of the PVL lesions associated with mild dysplasia and HPV-16 infection.

## Background

Proliferative verrucous leukoplakia (PVL) is a distinct variant of the oral leukoplakia, which is classified as an oral potentially malignant disorder (OPMD) and typified by a high transformation rate and morbidity [1, 2]**.**

PVL was first described in 1985 by Hansen et al. as a long-term progressive condition characterized by multifocal hyperkeratosis with confluent, exophytic, and proliferative manifestations [3].

The etiology of the PVL remains unknown. Tobacco use is not a common factor associated with PVL, being present in about a third of the cases [4, 5]. The relationship between the onset of PVL and alcohol consumption has not been established [6–9].

As regard the clinical management, there is lack of high-quality data to support the routine surgical excision of the non-invasive phase of PVL (Hansen’s grades 3–5) in an attempt to prevent future malignant transformation [2].

The role of the human papillomavirus (HPV) infection in the pathogenesis of the PVL is never been defined, and current evidences are contradictory [2, 9–11].

However, it should be considered that the infection of PVL lesions with high-risk HPV subtypes—such as HPV 16 and 18—could increase the transforming potential as a result of the expression of E6 and E7 viral oncoproteins, which functionally inactivate two human tumor suppressor proteins, p53 and pRb, respectively [12, 13]. Below, we present a case of PVL with HPV-16 infection managed by diode laser ablation 2 years and followed up after treatment.

## Case presentation

In January 2015, a 61-year-old Caucasian male was referred to our Oral Pathology and Medicine Unit by his own dentist for white oral lesions, since it was initially thought to be a fungal infection and he had received treatment based on topical antifungals, without a significant clinical improvement.

**Fig. 1:**
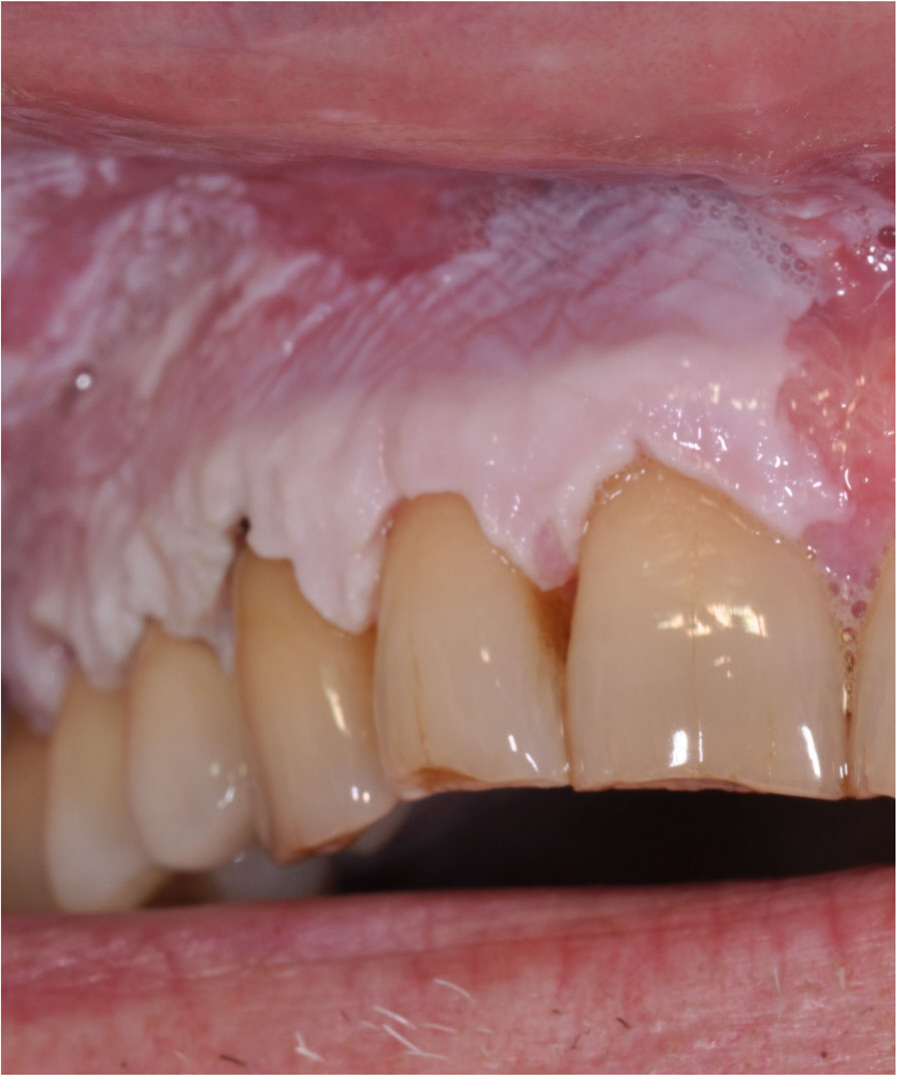
Verrucous hyperkeratosis in the right maxillary gingiva at the first clinical examination

Past medical history included appendectomy, cholecystectomy, and tonsillectomy. He had never smoked or used drugs recreationally and did not drink alcohol. At the time of the first visit, he was not taking any medication. No symptoms were referred by the patient, but he had noted a progressive increase of the lesion size in recent months. Clinical examination showed a thickened hyperkeratosis, which was confluent in widely exophytic papillary plaques. These lesions appeared homogeneously whitish-gray with verrucous surface and localized on the maxillary gingiva and palatal and alveolar ridge mucosa (Fig. 1). Incisional biopsies were performed, and multiple samples of tissue from the lesions were taken, using a 3-mm punch biopsy.

### Pathological findings

**Fig. 2:**
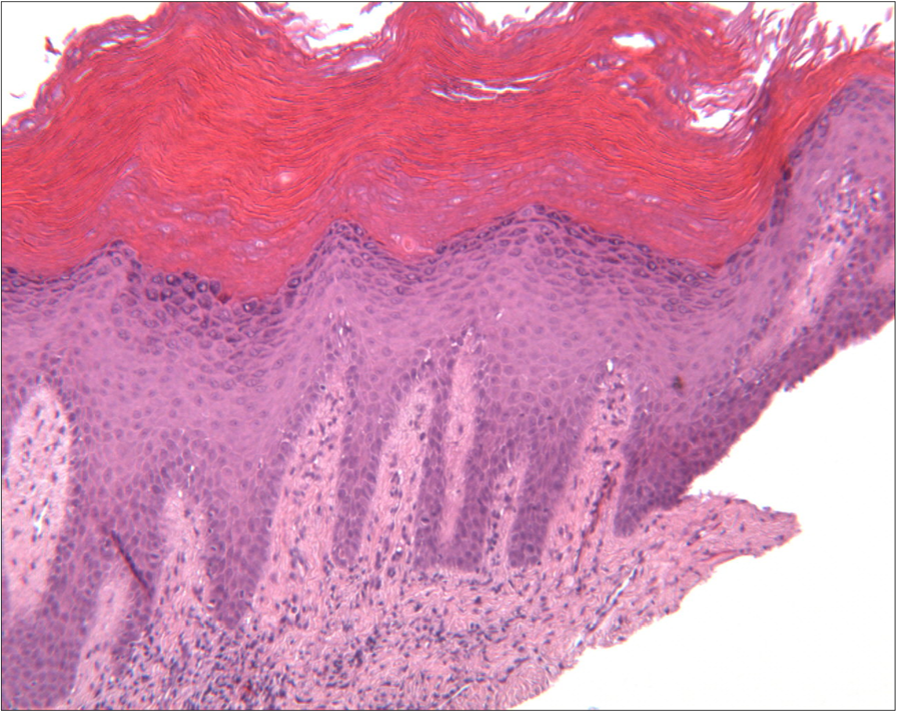
Severe verrucous hyperkeratosis with elongation of dermic ridges and mild chronic inflammatory infiltrate in the underlying stroma (H&E, 100 o.m)

**Fig. 3:**
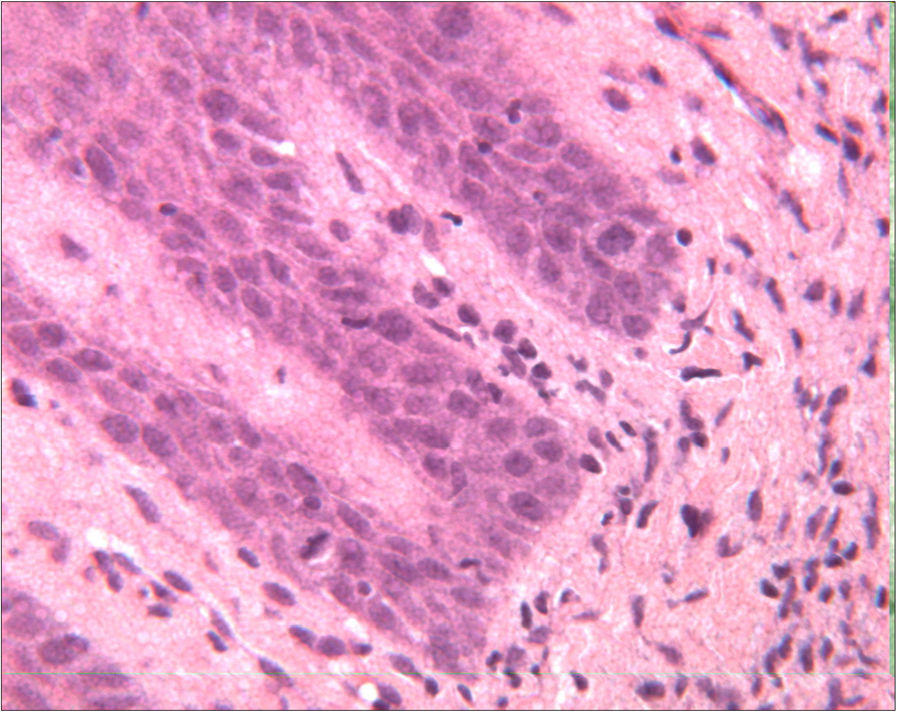
Magnification of the Fig. 2: proliferative verrucous leukoplakia, basal layer with mitosis and low grade cytological atypia (H&E, 400 o.m)

**Fig. 4:**
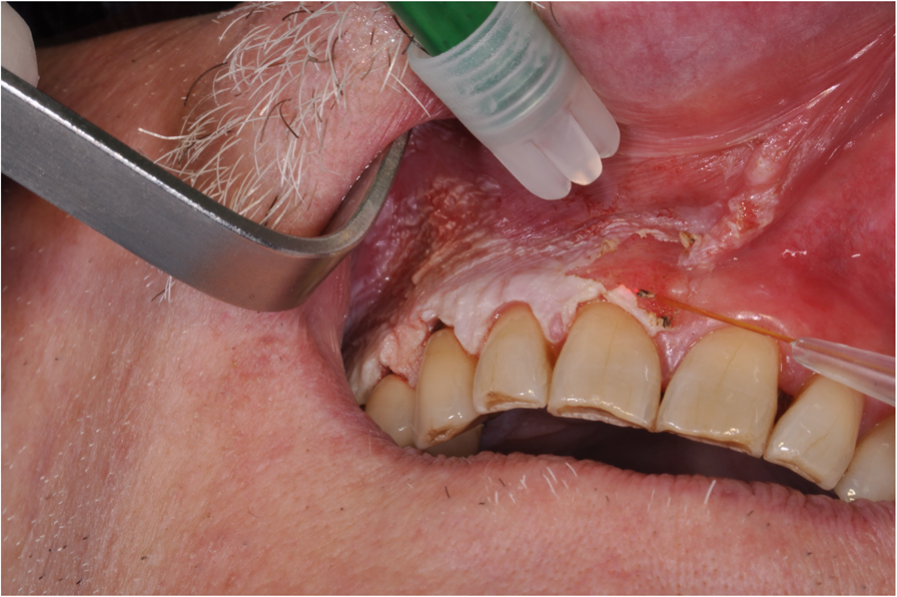
Surgical excision of the verrucous leukoplakia by a diode laser. Note the complete absence of hemorrhage

**Fig. 5:**
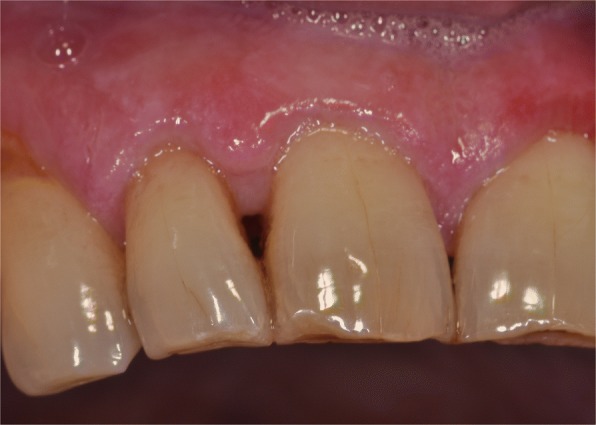
At 2 years of follow-up, the clinical examination showed a mild hyperkeratosis on the ipsilateral gingiva treated by a diode laser

**Fig. 6:**
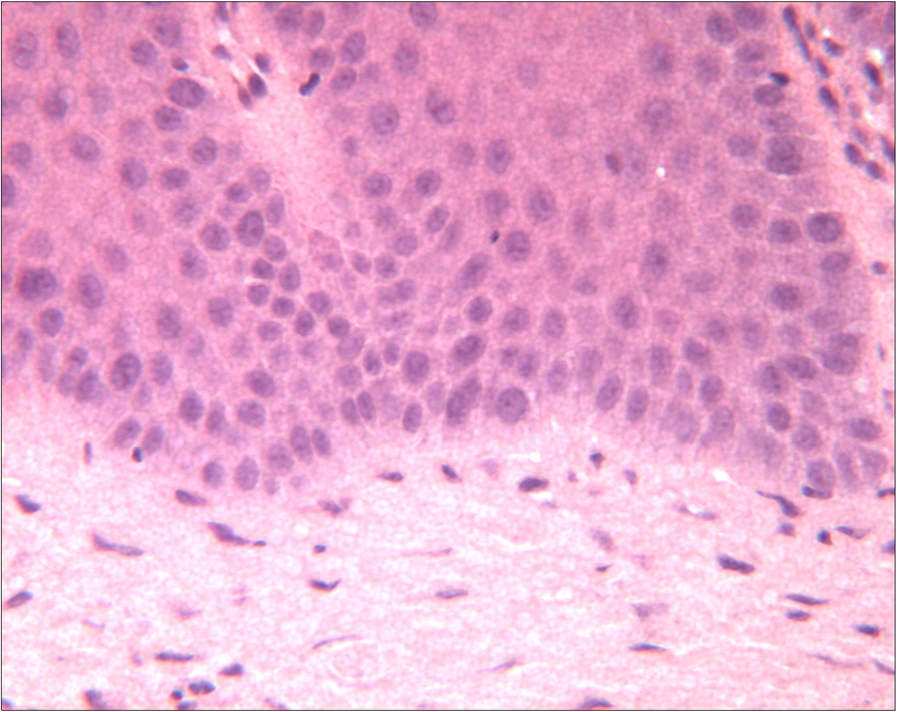
Sample of tissue taken in the same area as the previous laser treatment: proliferative verrucous leukoplakia, basal layer without evidence of mitosis and cytological atypia (H&E, 400 o.m)

The histopathological analysis revealed exophytic, hyperkeratotic lesions with prominent verruciform or papillary surface and acanthosis forming droplet-shaped epithelial projections into the lamina propria (Fig. 2). A sample was described as a hyperkeratosis with finding areas of verrucous hyperplasia. Other two gingival samples showed a hyperkeratosis with focal mild degree dysplasia (Fig. 3). Some areas showed intraepithelial alteration compatible with human papillomavirus infection. One portion of the gingival biopsy specimen, previously frozen, was used for DNA (deoxyribonucleic acid) extraction. Human papillomavirus DNA presence was confirmed with polymerase chain reaction (PCR) amplifications, and the viral typing, by direct sequencing of DNA common genomic region L1, displayed positive result for research of HPV subtype. Before surgical procedure, a complete blood cell count was performed to exclude coagulation disorders, along with viral profile. The excision of the lesion has been performed, under local anesthesia, using a diode laser (wavelength 808 nm) set at 1.5 W, the optical fiber, 300 μm, in a pulsated-wave emission mode to reduce the layer carbonization of the tissues (Fig. 4). The size of the lesion was 1.25 × 3.0 cm, and the laser settings were fluence 16 J/cm2, pulse length 20 ms, and spot diameter of 2 mm. No postoperative complications, as well as no swelling and/or pain reported by the patient, have been observed. A periodic follow-up every 6 months has been scheduled. At the last oral examination, 2 years after surgery, we found no signs of loss of alveolar process in correspondence of the surgical ablation site, whereas a mild hyperkeratosis of the maxillary vestibular gingiva was clinically objectified (Fig. 5). The microscopic examination performed on a tissue specimen taken in the same area of the laser treatment showed no signs of epithelial dysplasia (Fig. 6).

## Conclusions

Currently, no specific treatment modality has proven to be effective in the management of the PVL, either to slow down its progression toward malignant transformation and limit the high frequency of recurrence [2, 14]. Therefore, the patients with PVL need multiple biopsies over time to identify the histologic status of this progressive disorder [3].

The conventional treatment of PVL is surgical excision, although complete removal of the lesions may be challenging because of the multifocal manifestation of the oral lesions [14]. This approach can lead to significant morbidity and compromised quality of life. However, the presence of HPV-16 infection associated with epithelial dysplasia can justify—as the present case—the wide surgical excision of the affected site, trying to minimize the operational invasiveness. A systematic literature review found that there is a potentially causal association between oral leukoplakia infected by HPV 16 and epithelial dysplasia, as well as with oral squamous cell carcinoma (OSCC) [15]. It is important to consider that the biopsy sampling represents a powerful tool to demonstrate the relationship between HPV infection and oral dysplasia, since it has been shown that HPV DNA in exfoliated cells is not associated with HPV DNA detection in OSCCs [13, 15]. Furthermore, the multifocal development of the PVL lesions draws attention to the risk of field cancerization model, making the field mapping mandatory during the course of multiple biopsies sampling. The oncogenic potential of the HPV infection is attributable to its ability to insert specific DNA fragments of the early genes E5, E6, and E7 into the host cell genome. As a result of this integration, some key functions of tumor suppressor factors such as p21, p53, and pRb pathways are inhibited, leading to defects in apoptosis, DNA repair mechanisms, cell cycle regulation, and finally to [16]. In the current case, the choice of the laser excision of the whole dysplastic lesion was made in the attempt of achieving a viral contamination abatement through a minimally invasive procedure. As a matter of fact, the lateral expansion of the viral particles into the scar tissue maintains the infectivity in the tissue repair and permits the viral replication cycle, whereas the lack of activity of late proteins in the cells holds the primary infection in the basal layer, permitting recurrent infections [17]. It seems likely that the use of the laser surgery can induce—through the photo thermal effect—denaturation of the virus proteins in infected cells, extending the heat achieved by the high temperatures to areas beyond the lesion margins, thereby reducing the amount of infected tissue [18]. These findings have been confirmed by the lower viral load values in samples obtained at 20 days after surgery when using the diode laser [19]. Additionally, as regard the decision-making process in the surgical treatment of PVL with low grade dysplasia, it should be considered that no clinical characteristics—clinical appearance, lesion color, lesion surface—showed any significant correlation with malignant transformation to detect oral cancer in the early stage [20]. As previously reported, the tongue and gingiva are the most common transformation sites in PVL lesions, and it has been emphasized, in particular, the importance of the gingival surveillance, as described in the present case [6, 21]. Taking into account the high recurrence rate in the clinical course of PVL, the abovementioned surgical management was primarily directed to keeping under control the viral load of HPV infection and the potential progression of epithelial dysplasia to malignant transformation. Finally, particular vigilance should be turned to the periodontal status of PVL patients, since it is more likely that OSCCs in PVL areas are located in the masticatory mucosa, particularly in the gingiva. Moreover, periodontal disease has been associated with a small, but statistically significant, increase in overall oral cancer risk, which persisted among non-smokers [6, 22–25].

Although recurrence and progression are very common in the PVL lesions, whether the microscopic evaluation of the tissue sample confirms the HPV infection associated with mild dysplasia, the laser excision can be considered as surgical option. Biopsy mapping and complete laser excision of PVL tissue associated with epithelial dysplasia appear to be reliable, reproducible procedures associated with low complications and morbidity rates in terms of preventing malignant transformation. Strict follow-up is recommended for patients with PVL associated with HPV-16 infection and low grade dysplasia to detect oral cancer at an early stage.

Nonsurgical therapeutic approaches for PVL have been considered such as external beam radiation therapy, cryotherapy, topical vitamin, and topical chemotherapy, but with little success. Cryosurgery, photodynamic therapy, and antiviral methisoprinol therapy have also been suggested with significant benefit. In the future, anti-HPV, anti-TGF, and pro-apoptotic management strategies may be assessed [2, 4, 14].

## Abbreviations

DNA: Deoxyribonucleic acid
HPV: Human papillomavirus
OPMD: Oral potentially malignant disorder
OSCC: Oral squamous cell carcinoma
PCR: Polymerase chain reaction
PVL: Proliferative verrucous leukoplakia
